# Functional characterization of the GWAS lead SNP rs888663 and effects of *GDF15* SNPs on GDF15 levels in gestational hypertension and preeclampsia

**DOI:** 10.1007/s11033-026-11629-w

**Published:** 2026-03-07

**Authors:** Daniela A. Pereira, Julyane N. S. Kaihara, Luis Fernando P. Passeti, João Locke F. Araújo, Renan P. Souza, Ana C. Palei, Ricardo C. Cavalli, Chengyu Deng, Nadav Ahituv, Valeria C. Sandrim, Marcelo R. Luizon

**Affiliations:** 1 Graduate Program in Genetics, Institute of Biological Sciences, Federal University of Minas Gerais, Belo Horizonte, Minas Gerais Brazil; 2 Department of Biophysics and Pharmacology, Institute of Biosciences, Universidade Estadual Paulista (UNESP), Botucatu, São Paulo, Brazil; 3 Department of Genetics, Ecology and Evolution, Institute of Biological Sciences, Federal University of Minas Gerais, Belo Horizonte, Minas Gerais Brazil; 4 Instituto René Rachou, Fundação Oswaldo Cruz, Belo Horizonte, Minas Gerais Brazil; 5 Department of Surgery, University of Mississippi Medical Center, Jackson, MS USA; 6 Department of Gynecology and Obstetrics, Ribeirao Preto Medical School, University of Sao Paulo, Ribeirao Preto, São Paulo, Brazil; 7 Department of Bioengineering and Therapeutic Sciences, University of California, San Francisco, San Francisco, CA 94158 USA; 8 Institute for Human Genetics, University of California, San Francisco, San Francisco, CA 94143 USA

**Keywords:** Enhancer, Genetic polymorphisms, Gestational hypertension, Growth differentiation factor 15 (GDF15), Haplotypes, Preeclampsia

## Abstract

**Background:**

Growth differentiation factor 15 (GDF15) is a biomarker for cardiovascular diseases, and its circulating levels are altered in preeclampsia (PE), which shares mechanisms with cardiovascular diseases. Several SNPs in the *GDF15* locus were associated with GDF15 levels, including the GWAS lead SNP rs888663. However, no previous study had performed a functional characterization of rs888663 nor had tested the hypothesis that noncoding *GDF15* SNPs affect GDF15 levels in PE or gestational hypertension (GH). We examined whether variation of rs888663 affects its enhancer activity, whether genotypes and haplotypes of rs888663 and rs1059369 are associated with PE or GH, and their effects on GDF15 levels in healthy pregnant (HP) women, and in patients with PE and GH.

**Methods and results:**

We studied 233 HP women, 188 patients with PE, and 197 patients with GH. We performed a dual-luciferase reporter assay testing both rs888663 alleles (G/T), which found that the rs888663 region is a functional enhancer, and the T allele had a significantly higher enhancer activity. Genotypes were determined by Taqman allele discrimination assays. Plasma GDF15 levels were measured by ELISA. GDF15 levels were lower in PE and GH than in HP (*P* < 0.01). Patients with PE and GH carrying the TT genotype for rs1059369 and ‘T, T’ haplotype showed lower GDF15 levels than HP women carrying the same genotype and haplotype, respectively (*P* < 0.05).

**Conclusion:**

Our findings identified a novel candidate enhancer of *GDF15*, with the rs888663 T allele leading to increased enhancer activity, and suggest that *GDF15* SNPs affect GDF15 levels in PE or GH.

**Supplementary Information:**

The online version contains supplementary material available at 10.1007/s11033-026-11629-w.

## Introduction

Hypertensive disorders of pregnancy (HDP) are a major cause of maternal and perinatal morbidity and mortality and affects approximately 10% of all pregnancies [1, 2]. The prevalence of gestational hypertension (GH) and preeclampsia (PE) are 1.8–4.4% and 0.2–9.2%, respectively [2]. GH is defined as systolic blood pressure ≥ 140 mmHg and/or diastolic blood pressure ≥ 90 mmHg after 20 weeks of gestation occurring on two or more occasions at least 4 hours apart and in a woman who was normotensive prior to pregnancy. PE is defined as new-onset hypertension that can be accompanied by proteinuria, thrombocytopenia, impaired liver function, new-onset headache, pulmonary edema, or renal insufficiency [3].

The etiology of PE is not fully elucidated. However, circulating factors released from the placenta due to hypoxic and oxidative stress insults have been proposed to cause systemic inflammation and generalized maternal vascular endothelial dysfunction [4, 5]. Furthermore, PE is associated with an increased risk of future cardiovascular diseases, thereby indicating that PE and cardiovascular diseases might share common pathophysiological mechanisms [6–8].

Growth differentiation factor-15 (GDF15) is a stress-induced cytokine member of the transforming growth factor beta family. GDF15 is expressed in several tissues, including the placenta, being released by endothelial cells after stimulation with pro-inflammatory cytokines, and it is also associated with hypoxia and oxidative stress [9, 10]. Notably, GDF15 was suggested as a potential biomarker and therapeutic target for cardiovascular diseases, since it plays a cardioprotective role in the adult heart [9]. Considering its link with cardiovascular diseases and the increased placental expression of *GDF15*, it is relevant to examine the potential of GDF15 as a circulating biomarker for PE.

Circulating levels of GDF15 may vary due to pathophysiological conditions, but genetic polymorphisms can explain a significant portion of its inter-individual variability [11], which highlight the relevance of SNPs and regulatory regions that may affect *GDF15* expression. In this regard, a meta-analysis of GWAS showed several SNPs associated with GDF15 blood concentration at the *GDF15* locus [12], and the intergenic rs888663 SNP located upstream of *GDF15* [13] was the top SNP found to be associated with circulating GDF15 levels [12]. Another meta-analysis of GWAS identified the rs1059369 SNP to be associated with GDF15 levels [14]. While these SNPs may affect GDF15 levels, no previous study had performed a functional characterization of the GWAS lead SNP rs888663. Moreover, it is unknown whether *GDF15* SNPs are associated with HDP, and whether patients with GH and PE carrying different genotypes and haplotypes for these *GDF15* SNPs have different GDF15 levels.

In this study, we searched the ENCODE data for markers associated with enhancers and/or promoters located at the rs888663 SNP region and performed a dual-luciferase reporter assay with our candidate sequence and vectors containing either the reference (G) or alternative (T) alleles of rs888663. Moreover, we compared the GDF15 levels between healthy pregnant (HP) women and patients with PE and GH. We further examined whether rs888663 and rs1059369 are associated with HDP and the effects of their genotypes and haplotypes on GDF15 levels in PE or GH.

## Materials and methods

### Study population

All volunteers were enrolled in the Department of Obstetrics and Gynecology of the Ribeirao Preto Medical School of the University of Sao Paulo, and approval for the use of human subjects was obtained from the Institutional Review Board (#4682/2006). We studied women with uncomplicated pregnancies (HP, *n* = 233), and with PE (*n* = 188) and GH (*n* = 197), which were defined according to guidelines from the American College of Obstetricians and Gynecologists [3]. Women with essential/chronic hypertension were not included.

After obtaining written informed consent, maternal venous blood samples were collected, genomic DNA was extracted from the cellular component of 1 mL of whole blood by a salting-out method and stored at -20 °C until analyzed.

### Genotype determination

Genotypes for *GDF15* SNPs were determined using Taqman Allele Discrimination Assays (Applied Biosystems, Carlsbad, CA, USA): rs888663 (Assay ID: C___2036279_20), and rs1059369 (Assay ID: C___7494817_20). Real-time PCR was performed in a total volume of 12 µl (3 ng of template DNA, 1x TaqMan genotyping master mix, and 1x TaqMan allele discrimination assay) placed in 96-well PCR plates. Thermal cycling was performed in standard conditions, fluorescence was detected using StepOne Plus Real-Time PCR equipment (Applied Biosystems, Carlsbad, CA, USA), and results were analyzed with manufacturer’s software.

### Enzyme immunoassay for GDF15 measurement

Plasma GDF-15 concentrations were assessed using Human GDF-15 DuoSet ELISA kit (Catalog#DY957, R&D Systems, Minneapolis, MN, USA), following the manufacturer’s instructions. Samples were diluted at a 1:100 ratio, and the assay range was from 7.8 to 500 pg/mL.

### Selection of the candidate region

A region upstream the *GDF15* locus containing the rs888663 G > T SNP and several markers associated with promoters/regulatory regions were selected for the reporter assay (Supplementary Fig. 1). These markers include the histone H3 lysine 4 trimethylation (H3K4Me3), histone H3 lysine 4 mono-methylation (H3K4Me1), and histone H3 lysine 27 acetylation (H3K27Ac); overlap with transcription factors (TFs) binding sites, DNase I hypersensitivity sites, integrative analysis of ChIP-seq data for transcriptional regulators from GEO, ArrayExpress, and ENCODE, a distal enhancer-like signature according to ENCODE Registry of candidate cis-Regulatory Elements (cCREs; [15]); and GeneHancer [16] enhancer/promoter identifier (GH19J018372), and a mark for regulatory elements and gene interactions (Supplementary Fig. 1).

### Cloning of the candidate region

The 1357 bp candidate region location is chr19:18373265–18,374,622 (according to GRCh38). Amplification was done from genomic DNA using Q5 Ultra II DNA polymerase (M0544X, NEBNext^®^), gel purified and cloned into pGL4.23 [*luc2*/minP] luciferase vector. Primers for amplification are shown in Supplementary Table 1.

To access the effect of rs888663 SNP on the reporter gene expression, we used the Q5 site-directed mutagenesis kit (E0554S, NEBNext^®^) to perform a mutagenesis assay to replace the T allele to G allele using the plasmid we cloned as a template and the following primers for amplification are shown in Supplementary Table 1.

### Cell culture and transfection

HepG2 cell line (UCSF Cell and Genome Engineering Core) was grown in Eagle’s Minimum Essential Medium (10128-214, VWR), supplemented with 10% FBS (89510-194, VWR) and 1% Glutamax (35050061, Fisher Scientific), in a 5% CO_2_ incubator at 37 °C. For the luciferase assays, the cells were seeded into a 24-well plate at 5.8 × 10^4^ cells/well and transfected when they reached 80% confluency with 25 µl of the FuGENE^®^ 6 (E2693, Promega) /DNA/Opti-MEM^®^ (31985070, Fisher Scientific) mix.

Mix was prepared following manufacturer’s guidelines using 6µL of FuGENE^®^, 6 transfection reagent (Ratio Reagent: DNA 3:1), 1.886.7ng of plasmid DNA (pGL4.23[*luc2*/minP] reference allele, pGL4.23 alternative allele, 1.874.4ng of empty pGL4.23 (Promega) or 1.894.4ng of pGL4.13[*luc2*/SV40](Promega), plus 124.89ng of pGL4.74[hRluc/TK] (Promega) and the amount of Opti-MEM^®^ needed to make the final volume of 100 µl. Cells were lysed with 1x passive lysis buffer 24–48 h after transfection and measured for Firefly and *Renilla* luciferase activity using the Dual-Luciferase Reporter Assay System (E1980, Promega) in a GloMax 96 microplate Dual Injector Luminometer (Promega) following the manufacturer’s protocol. The empty pGL4.23 vector was used as a negative control and the pGL4.13 vector, containing a strong enhancer SV40, as the positive control. The dual luciferase assay is performed by sequentially measuring the Firefly and Renilla luciferase activities of the same sample, with the results expressed as the ratio of Firefly to Renilla luciferase activity.

### Statistical analysis

Demographics, clinical characteristics, and GDF15 levels of HP, and patients with PE and GH were compared by Student’s unpaired t-test, one way ANOVA, Mann-Whitney U test, or χ2 as appropriate, and reported as mean ± s.e.m. The effects of genotypes and haplotypes on GDF15 levels within each group were compared by analysis of variance followed by the Tukey test (normally distributed variables) or Kruskall-Wallis test followed by Dunn’s Multiple Comparison test (not normally distributed variables). The distribution of SNP genotypes in each study group was assessed for deviation from the Hardy–Weinberg equilibrium using exact tests, as described elsewhere [17], and differences in genotypes and allele frequencies were assessed using χ2 tests. The value of *P* < 0.05 was considered significant. The power to detect genotype effects on median GDF15 levels for the subgroups of PE and GH subjects was calculated by using the website: http://www.stat.ubc.ca/~rollin/stats/ssize/n2.html.

Haplotype frequencies were estimated by using the Haplo.stats package version 1.9.3 (http://cran.r-project.org/web/packages/haplo.stats/index.html) [18]. The possible haplotypes including the alleles of the two *GDF15* SNPs rs888663 (G > T) and rs1059369 (T > A) were: ‘T, T’, ‘T, A’, ‘G, T’ and ‘G, A’. However, the ‘G, A’ haplotype was excluded from the analysis due to its low frequency. Differences in haplotype frequencies were tested using χ2 tests and applied Bonferroni’s correction to correct for the number of comparisons made. A *P* value of *P* < 0.0125 (0.05/4, the number of possible haplotypes) was considered significant. Linkage disequilibrium (LD) was assessed by calculating D′ using the Haploview software (version 4.2; http://www.broad.mit.edu/mpg/haploview/) [19]. Data retrieved from the 1000 Genomes Phase III study for Europeans (CEU, Utah Residents with Northern and Western European Ancestry), East Asians (JPT, Japanese in Tokyo, Japan) and Africans (YRI, Yoruba in Ibadan, Nigeria) [20, 21] were also used for LD analysis.

Linear regression analysis was performed using the RStudio integrated development environment (IDE) (https://posit.co/products/open-source/rstudio/) to assess univariate relations between variables. In addition, a logistic regression was performed to assess the potential confounding influence of each covariate on the relationship between *GDF15* genotypes in the PE and GH groups. The variables of clinical importance were included in the multiple linear or logistic regression models. PE and GH were considered as dependent variables. Genotypes of *GDF15* SNPs, age, ethnicity, body mass index (BMI), primiparity, and gestational age at sampling were considered as independent variables.

Average readings of each sample (three replicates with three wells per plasmid) was used to calculate the relative firefly luciferase/Renilla luciferase activity. To calculate the fold-enrichment in relative luciferase activity, the relative luciferase activity of the positive control (pGL4.13) and each one of the experimental constructs, containing either the G or T allele of the rs888663 SNP, was normalized by the relative luciferase reading of the empty pGL4.23. Mann–Whitney U test (not normally distributed variables) was used to assess the differences between the groups and between each group, separately, and the negative control. A *P* value < 0.05 was considered statistically significant. The statistics analysis was run using GraphPad Prism 5.0 software (GraphPad, San Diego, CA).

## Results

### Identification of a novel candidate enhancer of *GDF15*

We performed an in-silico analysis to search for an enhancer candidate region in the *GDF15* locus including the SNP rs888663, which could affect *GDF15* expression. The genomic region surrounding this SNP was selected for the reporter assay, as it contains several markers associated with active regulatory elements (Supplementary Fig. 1), such as histone marks (H3K4me3, H3K4me1 and H3K27ac), TFs binding sites, DNase I hypersensitivity sites, ChIP-seq data for transcriptional regulators, a GeneHancer [16] enhancer/promoter regulatory region (H19J018372) and an enhancer-like signature according to cCREs [15].

**Fig. 1:**
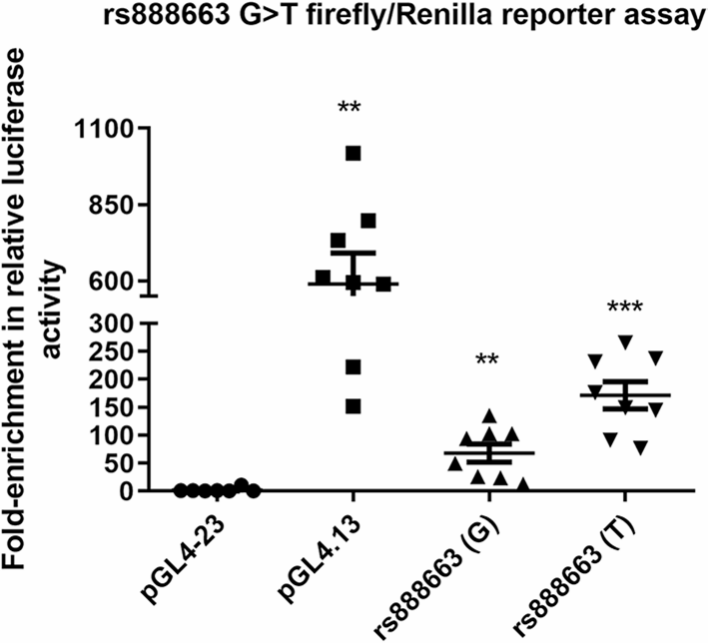
Fold-enrichment in relative luciferase activity measured in HepG2 cell line. The average readings of each sample were used to calculate the relative firefly luciferase/Renilla luciferase activity. The fold-enrichment was expressed as the ratio of firefly to Renilla luciferase activity normalized to the activity of the empty vector (pGL4.23). A vector containing a strong enhancer SV40 was used as positive control (pGL4.13). Differences between each group and the negative control were tested by Mann–Whitney U test. Data are expressed as mean + s.e.m. *p value < 0.05 compared to the empty vector

The enhancer candidate region was then cloned into a pGL4.23[*luc2*/minP] luciferase vector and transfected into a human hepatocyte cell line (HepG2) and their luciferase activity was measured and quantified. We used HepG2 cells, as GDF15 is widely expressed in various tissues with the highest levels being in the liver and placenta [22, 23]. Cells were co-transfected with either allele along with pGL4.74[hRluc/TK] vector expressing Renilla luciferase as an internal control to correct for transfection efficiency. In addition, we used the empty vector (pGL4.23) as a negative control and pGL4.13 as a positive control, which contains a strong SV40 enhancer/promoter. The relative Firefly luciferase/Renilla luciferase activity was calculated using the average readings of each sample. The positive control pGL4.13 had higher luciferase activity compared to negative control (pGL4.23), and both constructs with rs888663 G or T allele (*p* < 0.05). When comparing the relative luciferase activity of the G or T allele, the T allele had higher activity than both the G allele and the negative control (*p* < 0.05). The G allele only had higher activity when compared to negative control (*p* < 0.05). When analyzing the fold enrichment in relative luciferase activity, the pGL4.13 vector had an average of > 598.43-fold activity over the empty vector. The G allele had an average of > 68.06-fold activity over the empty vector, and the alternative allele (T) had an average of > 171.14-fold change activity over the empty vector (all *p* < 0.05) (Fig. 1).

### *GDF15* SNPs affect GDF15 levels in hypertensive disorders of pregnancy

The demographic and clinical characteristics of subjects enrolled in this study are shown in Table 1. PE, GH, and HP women showed similar ethnicity (% white), % current smoking, heart rate, hemoglobin, hematocrit and % primiparity (all *P* > 0.05). Patients with PE and with GH were older than HP and presented higher BMI and fasting glucose (*P* < 0.05). As expected, patients with PE and GH presented higher systolic and diastolic blood pressures than HP women (both *P* < 0.05), even though most of these patients were receiving antihypertensive therapy. Lower gestational age at delivery and lower newborn weights were found in patients with PE and GH than in HP women (all *P* < 0.05). When compared with GH, patients with PE presented higher proteinuria, systolic and diastolic blood pressures, and creatinine; and lower BMI, fasting glucose, newborn weights, and gestational age at delivery and at sampling (all *P* < 0.05). Univariate linear regression showed age, gestational age at sampling and BMI during pregnancy independently associated with PE and GH compared to HP women (*P* < 0.05 and OR > 1) and primiparity associated with GH compared to HP women (*P* < 0.05 and OR < 1; Supplementary Table 2).

**Table 1:** Clinical and demographic characteristics of the study subjects

Parameters	Healthy pregnant (*n* = 233)	Preeclampsia (*n* = 188)	*p* ^a^	Gestational hypertension (*n* = 197)	*p* ^b^	*p* ^c^
Age (years)	24.6 ± 0.4	27.2 ± 0.5	**0.000**	27.3 ± 0.5	**0.000**	0.981
Ethnicity (% White)	67.14	70.58	0.754	76.78	0.397	0.637
Current smokers (%)	11.11	8.65	0.504	12.44	0.759	0.320
BMI (Kg m–2) before pregnancy	23.3 ± 0.3	27.6 ± 0.7	**0.000**	30.4 ± 0.7	**0.000**	**0.007**
BMI (Kg m–2) during pregnancy	28.0 ± 0.3	32.6 ± 0.5	**0.000**	34.0 ± 0.5	**0.000**	0.053
SBP (mmHg)	111.1 ± 0.8	140.7 ± 1.6	**0.000**	133.4 ± 1.3	**0.000**	**0.001**
DBP (mmHg)	71.8 ± 0.6	88.3 ± 1.0	**0.000**	83.2 ± 1.0	**0.000**	**0.000**
HR (beats per min)	82.0 ± 0.6	82.8 ± 0.7	0.247	81.7 ± 0.6	0.635	0.125
Fasting glucose (mg/dL)	75.3 ± 1.0	84.3 ± 1.8	**0.000**	87.2 ± 1.9	**0.000**	0.332
Hb (mg/dL)	11.9 ± 0.1	12.1 ± 0.2	0.246	11.9 ± 0.1	0.826	0.121
Hct (%)	35.7 ± 0.4	36.5 ± 0.4	0.282	35.6 ± 0.3	0.743	0.117
Creatinine (mg/dL)	0.7 ± 0.0	0.7 ± 0.0	0.536	0.6 ± 0.0	**0.013**	**0.000**
24 h Pr (mg per 24 h)	ND	1107 ± 128.3	**–**	157.2 ± 10.7	**-**	**0.000**
Primiparity (%)	50.0	41.4	0.322	36.3	0.084	0.560
GAD (weeks)	39.7 ± 0.1	36.1 ± 0.3	**0.000**	38.8 ± 0.2	**0.000**	**0.000**
Newborn weight (g)	3303 ± 33.5	2606 ± 70.6	**0.000**	3163 ± 43.4	**0.011**	**0.000**
GAS (weeks)	36.6 ± 0.3	34.1 ± 0.4	**0.000**	35.5 ± 0.4	0.320	**0.000**

Abbreviations: BMI, body mass index; DBP, diastolic blood pressure; GAD, gestational age at delivery; GAS, gestational age at sampling; Hb, hemoglobin concentration; Hct, hematocrit; HR, heart rate; ND, not determined (however, negative dipstick test); SBP, systolic blood pressure; 24-h Pr, 24-h proteinuria. Values are the mean ± s.e.m

*p*^*a*^, healthy pregnant vs. preeclampsia

*p*^*b*^, healthy pregnant vs. gestational hypertension

*p*^*c*^, preeclampsia vs. gestational hypertension

Significant *p* values are in bold

Genotypes and allele frequencies for the rs888663 and rs1059369 SNPs are shown in Table 2. Distribution of genotypes showed no deviation from Hardy–Weinberg equilibrium (all *P* > 0.05; data not shown). We found no significant associations of any alleles and genotypes of *GDF15* SNPs with PE or GH when compared to HP (all *P* > 0.05; Table 2). These data were confirmed by logistic regression analysis adjusted for independent variables for each of the studied SNPs in PE and GH (Supplementary Tables 3 and Supplementary Table 4, respectively). Finally, we also found no significant differences in the distribution of *GDF15* haplotypes between PE or GH and HP (all *P* > 0.05; Table 3).

**Table 2:** *GDF15* genotypes and allele frequencies in healthy pregnant, and patients with preeclampsia and gestational hypertension

	Genotype or allele	Healthy pregnant	Preeclampsia	OR (95% CI)	*p* ^a^	Gestational hypertension	OR (95% CI)	*p* ^b^
		(*n* = 185)	(*n* = 177)			(*n* = 182)		
*rs888663 G > T*	GG	9 (4.8%)	8 (4.5%)	1.000 (Reference)	**–**	6 (3.3%)	1.000 (Reference)	**–**
Genotypes	GT	48 (26%)	47 (26.6%)	1.102 (0.392–3.098)	1.000	49 (26.9%)	1.531 (0.506–4.634)	0.581
	TT	128 (69.2%)	122 (68.9%)	1.072 (0.401–2.870)	1.000	127 (69.8%)	1.488 (0.515–4.304)	0.597
	GG + GT	57 (30.8%)	55 (31.1%)	1.000 (Reference)	**–**	55 (30.2%)	1.000 (Reference)	**–**
	TT	128 (69.2%)	122 (68.9%)	0.988 (0.632–1.543)	1.000	127 (69.8%)	1.028 (0.659–1.604)	0.910
	GG	9 (4.9%)	8 (4.5%)	1.000 (Reference)	**–**	6 (3.3%)	1.000 (Reference)	**–**
	GT + TT	176 (95.1%)	169 (95.5%)	1.080 (0.407–2.866)	1.000	176 (96.7%)	1.500 (0.523–4.304)	0.600
Alleles	G	66 (17.8%)	63 (17.8%)	1.000 (Reference)	**–**	61 (16.8%)	1.000 (Reference)	**–**
	T	304 (82.2%)	291 (82.2%)	1.003 (0.685–1.468)	1.000	303 (83.2%)	1.078 (0.735–1.581)	0.770
		(*n* = 183)	(*n* = 176)			(*n* = 181)		
*rs1059369 T > A*	TT	110 (60.1%)	106 (60.2%)	1.000 (Reference)	**–**	107 (59.1%)	1.000 (Reference)	**–**
Genotypes	TA	63 (34.4%)	59 (33.5%)	0.972 (0.623–1.515)	0.910	63 (34.8%)	1.028 (0.663–1.595)	0.911
	AA	10 (5.5%)	11 (6.3%)	1.142 (0.465- 2.800)	0.822	11 (6.1%)	1.131 (0.461–2.773)	0.822
	TT + TA	173 (94.5%)	165 (93.8%)	1.000 (Reference)	**–**	170 (93.9%)	1.000 (Reference)	**–**
	AA	10 (5.5%)	11 (6.2%)	1.153 (0.477–2.788)	0.824	11 (6.1%)	1.119 (0.463–2.705)	0.826
	TT	110 (60.1%)	106 (60.2%)	1.000 (Reference)	**–**	107 (59.1%)	1.000 (Reference)	**–**
	TA + AA	73 (39.9%)	70 (39.8%)	0.995 (0.652–1.519)	1.000	74 (40.9%)	1.042 (0.686–1.584)	0.915
Alleles	T	283 (77.3%)	271 (77.0%)	1.000 (Reference)	**–**	277 (76.5%)	1.000 (Reference)	**–**
	A	83 (22.7%)	81 (23.0%)	1.019 (0.719–1.444	0.929	85 (23.5%)	1.046 (0.741–1.477)	0.860

Abbreviations: CI, confidence interval; *GDF15*, Growth differentiation factor 15 gene; OR, odds ratio

*p*^*a*^, healthy pregnant vs. preeclampsia

*p*^*b*^, healthy pregnant vs. gestational hypertension

**Table 3:** Haplotypes frequencies formed by *GDF15* SNPs in healthy pregnant, and patients with preeclampsia and gestational hypertension

Haplotype	Healthy pregnant (*n* = 356)	Preeclampsia (*n* = 350)	*p* ^a^	OR (95% CI)	Gestational hypertension (*n* = 362)	*p* ^b^	OR (95% CI)
*‘T*,* T’*	0.607	0.591	0.825	1.000 (Reference)	0.602	0.926	1.000 (Reference)
*‘T*,* A’*	0.221	0.231	0.937	1.068 (0.739–1.545)	0.235	0.878	1.063 (0.743–1.520)
*‘G*,* T’*	0.159	0.177	0.732	1.113 (0.741–1.670)	0.163	0.850	1.007 (0.672–1.509)
		Global-stat = 2.394, df = 3, *p* = 0.495			Global-stat = 1.121, df = 3, *p* = 0.772		

Abbreviations: CI, confidence intervals; *GDF15*, global-stat, global score statistics; Growth differentiation factor 15 gene; OR, odds ratio

*p*^*a*^, healthy pregnant vs. preeclampsia

*p*^*b*^, healthy pregnant vs. gestational hypertension

**Fig. 2:**
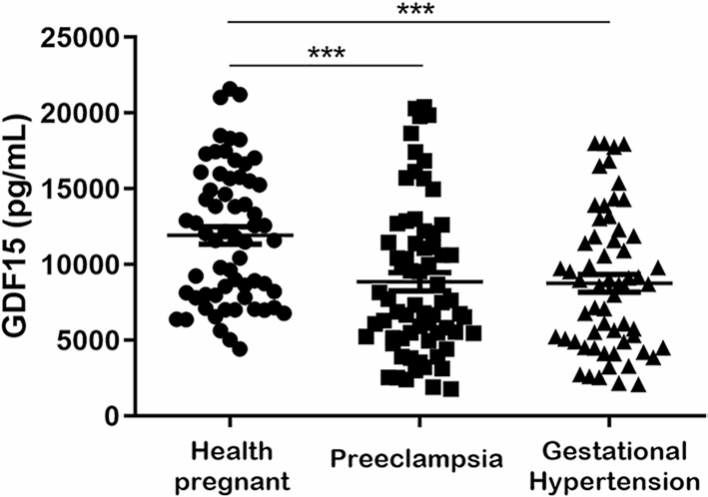
Plasma GDF15 concentrations in healthy pregnant women (*N* = 68), patients with preeclampsia (*N* = 72) and gestational hypertension (*N* = 63). The bars indicate the means ± s.e.m. ***P < 0.01 *versus* healthy pregnant women

Due to the nonavailability of plasma samples, we could not measure plasma GDF15 concentrations for all subjects enrolled in the study, which are shown in Fig. 2 for 68 HP women, 72 patients with PE, and 63 patients with GH. Notably, we found significant differences among the study groups, and PE and GH showed lower circulating GDF15 levels than HP women (both *P* < 0.05; Fig. 2). The clinical and demographic characteristics for the subjects whose plasma GDF15 levels were measured are shown in Supplementary Table 5, which showed similar values as compared to the whole group (Table 1), except for two differences: patients with PE were older than HP women (*P* < 0.05), but not older than GH (*P* > 0.05), and patients with PE presented lower newborn weights compared with HP and GH (all *P* < 0.05).

**Fig. 3:**
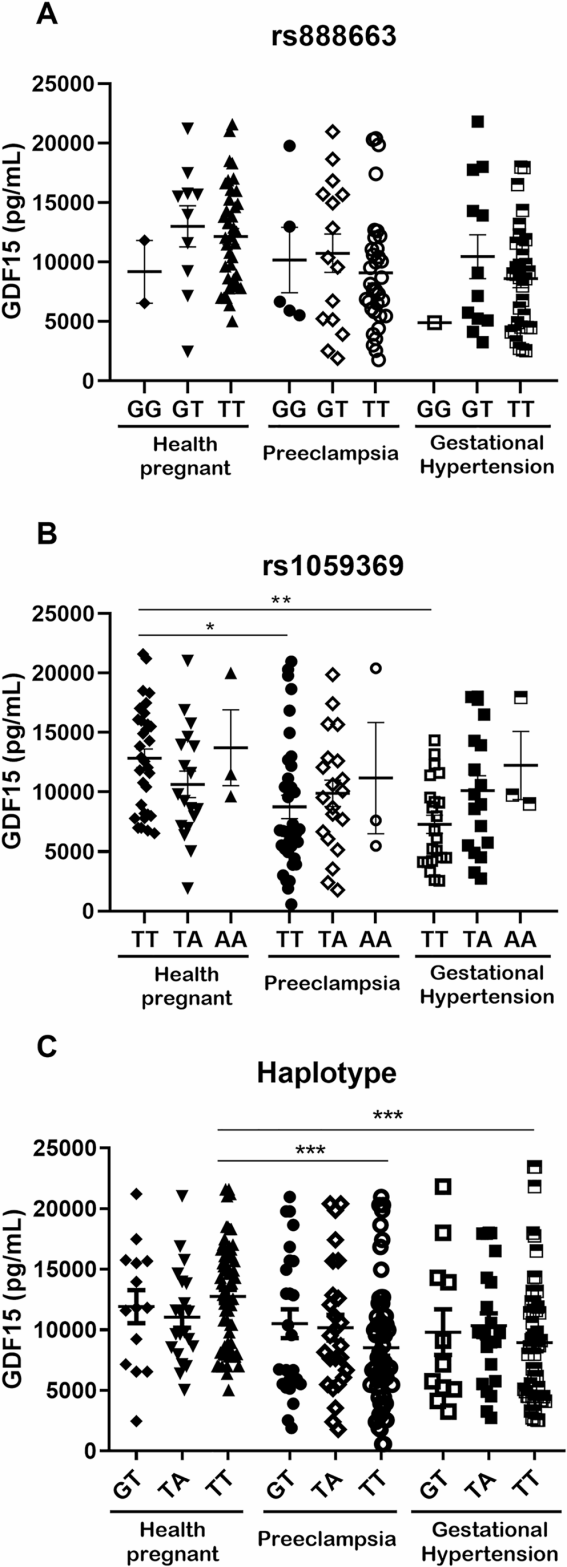
Plasma GDF15 concentrations in healthy pregnant women (*N* = 68), patients with preeclampsia (*N* = 72) and with gestational hypertension (*N* = 63) grouped according to the genotypes for the *GDF15* SNPs (**A**) rs888663 G > T, (**B**) rs1059369, T > A, and the (**C**) haplotypes formed by their alleles. The bars show the mean ± s.e.m. *P < 0.05 and **P < 0.01 vs. patients with preeclampsia and gestational hypertension carrying the TT genotype (**B**). ***P < 0.01 vs. patients with preeclampsia and gestational hypertension carrying the “T,T” haplotype (**C)**

We then examined the effects of *GDF15* genotypes and haplotypes on plasma GDF15 concentrations. We found no effects of the different genotypes for the rs888663 SNP (G > T) on plasma GDF15 levels (all *P* > 0.05; Fig. 3A). However, patients with PE and GH carrying the TT genotype for the rs1059369 SNP (T > A) showed lower GDF15 levels than HP women carrying the same genotype (both *P* < 0.05; Fig. 3B). We were able to detect the effects of the TT genotype for the rs1059369 SNP on GDF15 levels with a power of 0.90. Regarding haplotypes, patients with PE and GH carrying the ‘T, T’ haplotype showed higher plasma GDF15 levels than HP women with the same haplotype (both *P* < 0.05; Fig. 3C).

**Fig. 4:**
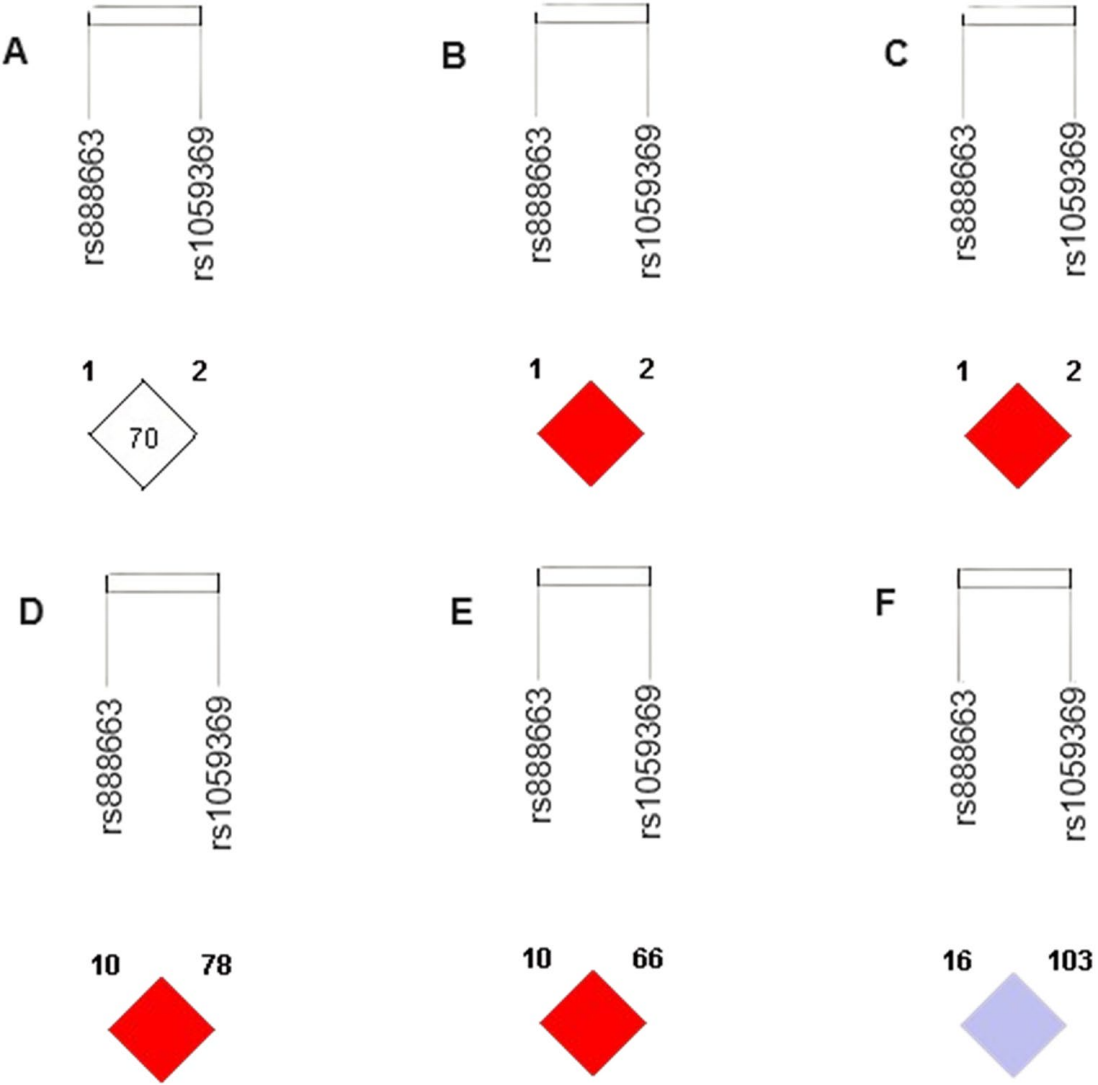
Linkage disequilibrium (LD) between the *GDF15* SNPs rs888663 and rs1059369 in healthy pregnant women (**A**), patients with preeclampsia (**B**) and gestational hypertension (**C**), and populations from the 1000 Genomes Project, namely Europeans (CEU, **D**), Japanese (JPT, **E**) and Africans (YRI, **F**). Intensity of LD is reflected in each box by color and numeric value. The number is D′ value (for example, 70 means D′ = 0.70). Color scheme: bright red, D’ = 1 and (LOD)’ ≥2; shades of pink/red, D < 1 and LOD’ ≥2; blue, D’ = 1 and LOD < 2; white, D < 1 and LOD < 2. LOD: Logarithm of odds

Next, we assessed the pairwise LD between the rs888663 and rs1059369 SNPs in the study groups (Fig. 4A–C), and for Europeans (CEU), Japanese (JPT) and Africans (YRI) populations from the 1000 Genomes Phase III study [19, 20] (Fig. 4D–F). While HP women showed weak LD (D’ < 1 and LOD < 2; Fig. 4A), we found a high LD (with D’ > 1 and LOD ≥ 2) between these SNPs in patients with PE (Fig. 4B) and GH (Fig. 4C), as well as in the CEU (Fig. 4D) and JPT (Fig. 4E) populations. Conversely, we found D′ = 1 and LOD < 2 in the YRI population (Fig. 4F), which may be explained by the demographic history and higher recombination events compared to non-Africans.

## Discussion

The main novel findings reported here are that (1) the region upstream the *GDF15 locus* and containing the rs888663 SNP presented enhancer activity and with the T allele showing significantly stronger activity than the G allele; (2) patients with PE and GH showed lower GDF15 levels than HP women; (3) patients with PE and GH carrying the TT genotype for the rs1059369 (T > A) and the ‘T, T’ haplotype showed lower GDF15 levels than HP women carrying the same genotype and haplotype, respectively.

In this study we hypothesized that SNPs located within enhancer regions of the *GDF15* locus modulate enhancer activity, leading to altered transcription of *GDF15*. We tested the identified candidate region for its *cis*-regulatory activity by using a dual-luciferase reporter assay [24]. The T allele of rs888663 showed higher luciferase activity compared to the G allele or negative control (empty vector), which provides evidence that the T allele can increase gene reporter expression when compared to the reference allele G. While further functional studies are needed to validate this information in vivo, our findings are in line with ENCODE data [25], which characterizes regulatory regions and shows that the SNPs rs888663 and rs1059369 overlaps with regions considered to be strong enhancers, and with previous GWAS studies showing the SNP rs888663 as associated with GDF15 and PGPEP1 levels and might affect their expression [12, 26]. Moreover, GTEx data [27] for rs888663 showed that the T allele is associated with increased expression of *GDF15*, *PGPEP1*,* DDX49*,* LRRC25* (in skeletal muscle) and of the long non-coding RNA AC005932.1, and the G allele is associated with decreased expression. However, the G allele is associated with increased expression of *LRRC25* in whole blood and esophagus [28], thus highlighting the individuality of regulatory sequences in different cell types and tissues.

Moreover, both rs888663 and rs1059369 are in strong LD with several other functional SNPs located within a potential regulatory region upstream of *GDF15* locus [13]. For example, the rs62122429 was previously found to be associated with GDF15 levels [28].Therefore, we assessed the pairwise LD between rs888663 and rs1059369 in our study groups and in CEU, JPT and YRI populations from the 1000 Genomes Project [20, 21]. We found that these SNPs are in complete LD in PE and GH, and in CEU and JPT populations. However, it is important to highlight that we did not examine other potential functional *GDF15* SNPs that could affect *GDF15* levels, and our findings need to be further replicated. It is critical to investigate the causative variants to better understand regarding the biological mechanisms underlying the regulation of gene expression [29]. In this context, reporter gene assays seem to be a good tool to identify a functional SNPs within an enhancer region. Notably, genetic factors account for up to 38% of the variability in GDF15 levels, and the rs888663 was the main SNP associated with GDF15 levels [26].

GDF15 levels increases following the trimesters of normal pregnancy. It can increase from 40-fold in the first trimester to 200-fold in the third trimester when compared to nonpregnant values [30]. Discordant findings have been reported regarding maternal circulating GDF15 levels in PE. While some studies showed elevated GDF15 levels in patients with PE compared to HP control in serum [31–33] and in plasma samples [34], other studies showed decreased serum GDF15 levels in patients with PE compared to HP control [35, 36], or they showed no differences in serum GDF15 levels when compared GH with HP [35]. In our study, patients with PE and GH presented lower GDF15 levels than HP. However, no previous study had found decreased levels of GDF15 in plasma from patients with HDP. Methodological issues need to be considered among studies, such as differences in the immunoassays used to assess GDF15 levels, and different gestational ages at sampling among studies. Therefore, further studies are needed to clarify the differences among studies, and to examine whether a reduction in GDF15 levels could either contribute to the development of PE or be a compensatory maternal response.

PE shares risk factors with cardiovascular diseases, including endothelial dysfunction and exacerbated inflammatory response [8], and GDF15 is upregulated in response to cell stress and injury [37]. Moreover, GDF15 was recently proposed as a biomarker for cardiovascular diseases and is a potential biomarker for PE [34, 38]. In this scenario, abnormal placentation results in disrupted levels of sFlt-1 and PlGF, primarily due to inadequate remodeling of the maternal spiral arteries and resulting placental ischemia. Under these hypoxic conditions, the placenta releases increased amounts of sFlt-1, which in turn significantly lowers PlGF levels due to inhibition by sFlt-1, as revised elsewhere [39].

Maternal levels of sFlt-1, PlGF, and their ratio (sFlt-1/PlGF) are key biomarkers for screening, diagnosing, and monitoring PE. A large study with suspected PE at 24 + 0 to 36 + 6 weeks’ gestation validated a sFlt-1/PlGF ratio ≤ 38 to effectively rule out PE within 1 week (99.3%; negative predictive value), and a ratio > 38 to rule in PE within 4 weeks (36.7%; positive predictive value) [40]. Another study explored adding GDF15 to improve sensitivity of the sFlt-1/PIGF ratio measured at 36 weeks’ gestation in identifying who are more likely to develop PE. While GDF15 or PlGF alone had 46.3% sensitivity, and sFlt-1/PlGF had 61%, the GDF15×sFlt-1/PlGF combination improved sensitivity to 68.3%, suggesting GDF15 could be used together with sFlt-1/PlGF ratio to improve PE prediction [34].

However, we have not examined the relationship between GDF15 levels and relevant risk factors for PE, including markers of hypoxia, oxidative stress, and endothelial dysfunction, such as SPARC, whose expression is time-dependent during placental development, suggesting that the presence of this protein may be linked to the processes of tissue invasion involved in placental formation [41]. In this context, further studies are also needed to evaluate the correlation of GDF15 levels with those markers in PE.

To our knowledge, no previous study has examined whether *GDF15* SNPs or haplotypes are associated with HDP. While we found no significant associations of rs888663 and rs1059369 and their haplotypes, case-control association studies in populations with different backgrounds are needed to rule out their role on susceptibility to PE and/or GH. Noteworthy, no previous study has examined the effects of rs888663 and rs1059369 SNPs of *GDF15* and their haplotypes on circulating GDF15 levels in HDP. Both *GDF15* SNPs were previously identified in GWAS to be associated with circulating GDF15 levels [12, 14, 26]. The rs888663 was the top significantly associated SNP with GDF15 levels in blood tissue [12], and both rs888663 and rs1059369 SNPs were associated with plasma GDF15 concentrations [14, 26]. Moreover, these SNPs were described to be eQTLs from the GTEx project for *GDF15* expression in different tissues [13, 27].

While the rs888663 SNP (G > T) did not alter plasma GDF15 levels, patients with PE and GH carrying the TT genotype of rs1059369 SNP (T > A) and the ‘T, T’ haplotype showed lower GDF15 levels than HP women carrying the same genotype and haplotype, respectively. Although we found no differences within the same group, the difference between groups for a given SNP genotype might be explained by the differences in GDF15 levels between groups, which were lower in PE and GH than in HP women. Moreover, this observation could also be due to the fact that the AA and TT genotypes of rs1059369 and rs888663 SNPs, respectively, are associated with higher GDF15 expression, according to the GTEx portal [27]. Further studies are required to confirm these hypotheses. Taken together, these findings confirm that *GDF15* SNPs have effects on GDF15 levels. However, these effects may vary under different physiological and/or disease conditions, and other factors may also affect *GDF15* expression independent of their genotypes.

In summary, altered enhancer activity can disrupt gene regulation, leading to abnormal cellular functions and disease [42, 43]. Enhancers regulate gene expression by recruiting transcription factors and coactivators, and their dysfunction can impact key biological pathways [44]. In pregnancy, changes in enhancer function may affect gene expression and disease susceptibility, such as altered GDF15 levels seen in PE that could influence maternal endothelial function, inflammatory responses, or trophoblast invasion [45], and higher GDF15 levels associated with vomiting, and hyperemesis gravidarum [46]. Further functional studies are needed to validate our findings; however, our study provides a biologically plausible framework that aligns with current understanding of the pathophysiology of HDP.

The present study has limitations. First, we could only study a relatively small number of patients and plasma GDF15 levels were determined in an even smaller number of patients. Despite this limitation, we found a significant difference in plasma GDF15 between groups of patients with HDP and controls, and we also found differences in the GDF15 concentrations when groups were separated according to different GDF15 genotypes and haplotypes. Second, we did not assess GDF15 levels in the placenta or other tissues; instead, our analysis was limited to total plasma GDF15 levels. Another limitation of our study is the lack of analysis correlating GDF15 levels and the studied SNP genotypes with gestational age at PE onset, which could add more clinical relevance to GDF15 as a potential biomarker. Future research with larger, prospectively stratified cohorts will be essential to fully elucidate the relationship between GDF15 and specific PE subtypes.

In addition, although reporter assays are a well-known tool for examining regulatory elements, those constructs transfected into cell lines do not replicate in vivo gene regulation. Reporter assays lack of distal enhancer elements, regulators and the effect of intronic elements, as well as effects of a specific nuclear location or genomic environment on transcription. Furthermore, the epigenetic history that might control a promoter and enhancer in vivo cannot be reproduced, neither the histone packaging nor the chromatin condensation [47]. However, due to the potential of GDF15 as an emerging biomarker for complex diseases, it is relevant to better understand the regulatory regions of *GDF15.* Notably, our novel findings provide for the translation of GWAS results into molecular basis for human diseases [48].

## Conclusions

We identified an enhancer candidate region upstream the *GDF15* locus, harboring the GWAS lead SNP rs888663 (G > T) associated to GDF15 levels in blood tissue, and validated this region by using a luciferase reporter assay showing that the T allele presents significantly stronger enhancer activity when compared to the G allele and the negative control. Furthermore, we observed lower GDF15 levels in patients with PE and GH compared to HP women. We provide novel evidence that patients with PE and GH carrying the TT genotype for the rs1059369 (T > A) SNP and the ‘T, T’ haplotype showed lower GDF15 levels than HP women. Our novel findings suggest that *GDF15* genotypes and haplotypes affect GDF15 levels in HDP and help to explain the regulation of *GDF15* expression, which can guide future studies focusing on its potential as a biomarker.

## Supplementary Information

Below is the link to the electronic supplementary material.

Supplementary Material 1

Supplementary Material 2

Supplementary Material 3

Supplementary Material 4

Supplementary Material 5

Supplementary Material 6

## Data Availability

The data that supports the findings of this study are available from the corresponding author, upon reasonable request.

## Abbreviations

eQTL: Expression quantitative trait locus
ENCODE: The Encyclopedia of DNA Elements
GDF15: Growth differentiation factor-15
GH: Gestational hypertension
GTEx: The Genotype-Tissue Expression project
GWAS: Genome-wide association study
HDP: Hypertensive disorders of pregnancy
HP: Healthy pregnant women
PE: Preeclampsia
PlGF: Placental growth factor
SNP: Single nucleotide polymorphism
sFlt-1: Soluble fms-like tyrosine kinase-1
