# Supplementary material for: Functional characterization of the GWAS lead SNP rs888663 and effects of GDF15 SNPs on GDF15 levels in gestational hypertension and preeclampsia
Source: Mol Biol Rep. 2026 Mar 7;53(1):476. doi: 10.1007/s11033-026-11629-w (PMC12967388; doi:10.1007/s11033-026-11629-w)

**SUPPLEMENTARY FIGURE 1**

**Supplementary Figure 1.** UCSC Genome Browser view of the enhancer candidate region upstream the *GDF15* locus. The cloned region is delimited by the red lines and the rs888663 polymorphism is highlighted in black. The selected enhancer candidate region overlaps with several ENCODE and GenHancer markers, including H3K4Me3, H3K4Me1, H3K27A, Transcription Factor binding sites, a distal enhancer-like signature according to cCREs, DNase I hypersensitivity sites, an enhancer/promoter identifier (GH19J018372) and a mark for regulatory elements and gene interactions.

Abbreviations: GDF15: Growth Differentiation factor 15; ENCODE: Encyclopedia of DNA elements; cCREs: candidate Cis-Regulatory Elements; H3K4Me3: Histone H3 lysine 4 trimethylation; H3K4Me1: Histone H3 lysine 4 mono-methylation; H3K27Ac: Histone H3 lysine 27 acetylation.


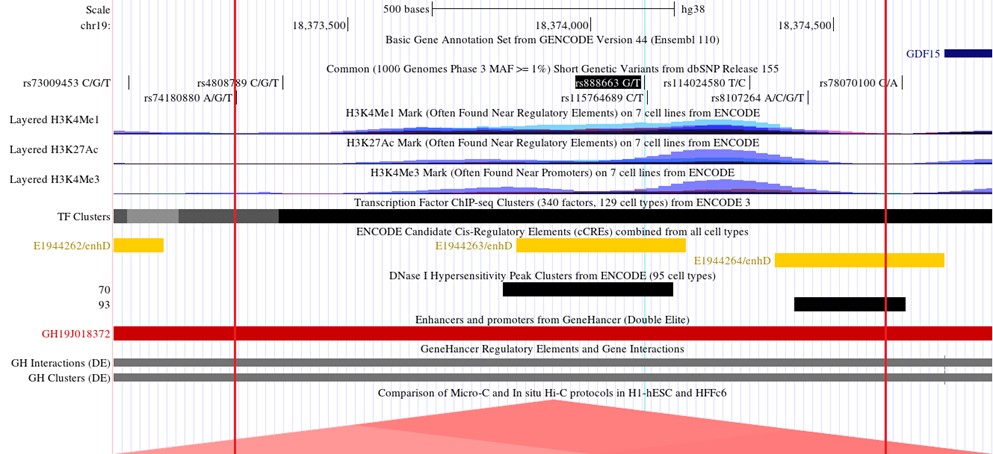

Supplement: Supplementary file 1 — Supplementary Material 1 [file 11033_2026_11629_MOESM1_ESM.docx]
