# Supplementary material for: Functional characterization of the GWAS lead SNP rs888663 and effects of GDF15 SNPs on GDF15 levels in gestational hypertension and preeclampsia
Source: Mol Biol Rep. 2026 Mar 7;53(1):476. doi: 10.1007/s11033-026-11629-w (PMC12967388; doi:10.1007/s11033-026-11629-w)
Supplement: Supplementary file 2 — Supplementary Material 2 [file 11033_2026_11629_MOESM2_ESM.docx]

**Supplementary Table 1.** Primers used for the enhancer candidate region amplification and for the mutagenesis assay.

| Target | Forward primer | Reverse primer |
| --- | --- | --- |
| *GDF15* enhancer region | 5’-CCTAACTGGCCGGTACCCTGAAGTCAGGAGTTTGAGACCAG-3’ | 5’-GGCCAGATCTTGATATCCGGCAGCTGAGTATGAGTCAGCAG-3’ |
| rs888663 (T>G) | 5’-CAGGGAGAGA**g**AGACACAAAG-3’ | 5’-GCTCTCCCTCCATTTCTG-3’ |
