# Supplementary material for: Functional characterization of the GWAS lead SNP rs888663 and effects of GDF15 SNPs on GDF15 levels in gestational hypertension and preeclampsia
Source: Mol Biol Rep. 2026 Mar 7;53(1):476. doi: 10.1007/s11033-026-11629-w (PMC12967388; doi:10.1007/s11033-026-11629-w)
Supplement: Supplementary file 3 — Supplementary Material 3 [file 11033_2026_11629_MOESM3_ESM.docx]

**Supplementary Table 2.** Univariate logistic regression in preeclampsia and gestational hypertension.

| *Logistic model PE* | *Estimate* | *Std. Error z* | *z value* | *Pr(>\|z\|)* | *OR (95% CI)* |  | *Logistic model GH* | *Estimate* | *Std. Error z* | *z value* | *Pr(>\|z\|)* | *OR (95% CI)* |
| --- | --- | --- | --- | --- | --- | --- | --- | --- | --- | --- | --- | --- |
| *(Intercept)* | -2.059 | 0.454 | -4.532 | **<0.001** | 0.128 (0.052-0.307) |  | *(Intercept)* | -1.930 | 0.444 | -4.348 | **<0.001** | 0.145 (0.060-0.342) |
| *Age (years)* | 0.071 | 0.017 | 4.178 | **<0.001** | 1.074 (1.039-1.111) |  | *Age (years)* | 0.070 | 0.017 | 4.172 | **<0.001** | 1.072 (1.038-1.110) |
| *(Intercept)* | 5.780 | 1.238 | 4.685 | **<0.001** | 330.1 (33.250- 4308.3) |  | *(Intercept)* | 2.430 | 1.040 | 2.334 | **0.020*** | 11.336 (1.595-97.1482) |
| *GAS (weeks)* | -0.163 | 0.034 | -4.728 | **<0.001** | 0.850 (0.792- 0.906) |  | *GAS (weeks)* | -0.065 | 0.029 | -2.269 | **0.023*** | 0.937 (0.884-0.989) |
| *(Intercept)* | -4.662 | 0.666 | -6.997 | **<0.001** | 0.010 (0.003-0.033) |  | *(Intercept)* | -5.916 | 0.721 | -8.203 | **<0.001** | 0.003 (0.001-0.010) |
| *BMI (kg/m²) during pregnancy* | 0.148 | 0.0220 | 6.740 | **<0.001** | 1.159 (1.112-1.212) |  | *BMI (kg/m²) during pregnancy* | 0.187 | 0.023 | 7.979 | **<0.001** | 1.206 (1.154-1.265) |
| *(Intercept)* | -13.57 | 535.41 | -0.025 | 0.98 | - |  | *(Intercept)* | <0.001 | 1.414 | 0.000 | 1.000 | - |
| *Ethnicity (White)* | 13.26 | 535.41 | 0.025 | 0.98 | - |  | *Ethnicity (White)* | -1.268 | 1.419 | -0.089 | 0.929 | - |
| *Ethnicity (mixed race)* | 13.23 | 535.41 | 0.025 | 0.98 | - |  | *Ethnicity (mixed race)* | -0.2963 | 1.435 | -0.206 | 0.836 | - |
| *Ethnicity (Black)* | 13.61 | 535.41 | 0.025 | 0.98 | - |  | *Ethnicity (Black)* | -0.3365 | 1.454 | -0.231 | 0.817 | - |
| *(Intercept)* | -0.029 | 0.140 | -0.210 | 0.834 | - |  | *(Intercept)* | 0.168 | 0.133 | 1.260 | 0.208 | - |
| *Primiparity (%)* | -0.340 | 0.207 | -1.632 | 0.103 | - |  | *Primiparity (%)* | -0.564 | 0.204 | -2.762 | **0.006**** | 0.569 (0.381-0.848) |
| *(Intercept)* | -0.251 | 0.504 | -0.499 | 0.618 | - |  | *(Intercept)* | -0.406 | 0.527 | -0.769 | 0.442 | - |
| *rs888663 (GT)* | 0.2513 | 0.544 | 0.462 | 0.644 | - |  | *rs888663 (GT)* | 0.426 | 0.565 | 0.754 | 0.451 | - |
| *rs888663 (TT)* | 0.187 | 0.520 | 0.359 | 0.719 | - |  | *rs888663 (TT)* | 0.398 | 0.542 | 0.734 | 0.463 | - |
| *(Intercept)* | 0.095 | 0.437 | 0.218 | 0.827 | - |  | *(Intercept)* | 0.095 | 0.437 | 0.218 | 0.827 | - |
| *rs1059369 (AT)* | -0.161 | 0.473 | -0.340 | 0.734 | - |  | *rs1059369 (AT)* | -0.095 | 0.472 | -0.202 | 0.840 | - |
| *rs1059369 (TT)* | -0.142 | 0.458 | -0.310 | 0.757 | - |  | *rs1059369 (TT)* | -0.123 | 0.458 | -0.269 | 0.788 | - |

Abbreviations: GAS, gestational age at sampling; CI, confidence intervals; OR, odds ratio; PE, preeclampsia; GH, gestational hypertension.

Significant *P* values are in bold.
