# Supplementary material for: Functional characterization of the GWAS lead SNP rs888663 and effects of GDF15 SNPs on GDF15 levels in gestational hypertension and preeclampsia
Source: Mol Biol Rep. 2026 Mar 7;53(1):476. doi: 10.1007/s11033-026-11629-w (PMC12967388; doi:10.1007/s11033-026-11629-w)
Supplement: Supplementary file 4 — Supplementary Material 4 [file 11033_2026_11629_MOESM4_ESM.docx]

**Supplementary Table 3.** Multivariate logistic regression analysis adjusted for independent variables in Gestational hypertension (GH).

| *Logistic model GH* | *Estimate* | *Std. Error z* | *z value* | *Pr(>\|z\|)* | *OR (95% CI)* |  | *Estimate* | *Std. Error z* | *z value* | *Pr(>\|z\|)* | *OR (95% CI)* |
| --- | --- | --- | --- | --- | --- | --- | --- | --- | --- | --- | --- |
| *(Intercept)* | -7.465 | 1.174 | -6.357 | **<0.001** | 0.001 (0.0001-0.005) | *(Intercept)* | -6.322 | 0.992 | -6.371 | **<0.001** | 0.002 (0.000-0.012) |
| *rs888663GT* | 0.792 | 0.774 | 1.023 | 0.306 | - | *rs1059369AT* | -0.463 | 0.539 | -0.860 | 0.3901 | - |
| *rs888663TT* | 0.831 | 0.750 | 1.109 | 0.268 | - | *rs1059369TT* | -0.469 | 0.519 | -0.905 | 0.3654 | - |
| *Age (years)* | 0.051 | 0.020 | 2.602 | **0.009**** | 1.052 (1.013-1.094) | *Age (years)* | 0.049 | 0.020 | 2.510 | **0.012*** | 1.050 (1.011-1.092) |
| *BMI (kg/m²) during pregnancy* | 0.172 | 0.025 | 6.960 | **<0.001** | 1.188 (1.134-1.249) | *BMI (kg/m²) during pregnancy* | 0.177 | 0.025 | 7.052 | **<0.001** | 1.193 (1.139-1.256) |
|  |  |  |  |  |  |  |  |  |  |  |  |
| *(Intercept)* | -6.049 | 1.056 | -5.726 | **<0.001** | 0.002 (0.0003-0.017) | *(Intercept)* | -5.045 | 0.871 | -5.789 | **<0.001** | 0.006 (0.001-0.034) |
| *rs888663GT* | 0.634 | 0.757 | 0.837 | 0.402 | - | *rs1059369AT* | -0.517 | 0.529 | -0.976 | 0.329 | - |
| *rs888663TT* | 0.687 | 0.731 | 0.939 | 0.348 | - | *rs1059369TT* | -0.496 | 0.507 | -0.977 | 0.329 | - |
| *BMI (kg/m²) during pregnancy* | 0.179 | 0.025 | 7.184 | **<0.001** | 1.196 (1.141-1.259) | *BMI (kg/m²) during pregnancy* | 0.183 | 0.025 | 7.229 | **<0.001** | 1.201 (1.145-1.265) |
| *Primiparity (Yes)* | -0.266 | 0.254 | -1.047 | 0.295 | 0.766 (0.465-1.262) | *Primiparity (Yes)* | -0.265 | 0.255 | -1.041 | 0.298 | - |
|  |  |  |  |  |  |  |  |  |  |  |  |
| *(Intercept)* | -6.356 | 1.030 | -6.169 | **<0.001** | 0.002 (0.0002-0.012) | *(Intercept)* | -5.304 | 0.850 | -6.242 | **<0.001** | 0.005 (0.001-0.025) |
| *rs888663GT* | 0.692 | 0.751 | 0.922 | 0.356 | - | *rs1059369AT* | -0.520 | 0.524 | -0.991 | 0.321 | - |
| *rs888663TT* | 0.723 | 0.726 | 0.996 | 0.319 | - | *rs1059369TT* | -0.501 | 0.503 | -0.996 | 0.319 | - |
| *BMI (kg/m²) during pregnancy* | 0.184 | 0.025 | 7.417 | **<0.001** | 1.202 (1.147-1.265) | *BMI (kg/m²) during pregnancy* | 0.188 | 0.025 | 7.453 | **<0.001** | 1.207 (1.151-1.271) |
|  |  |  |  |  |  |  |  |  |  |  |  |
| *(Intercept)* | -0.118 | 0.543 | -0.216 | 0.829 | - | *(Intercept)* | 0.321 | 0.451 | 0.712 | 0.476 | - |
| *rs888663GT* | 0.391 | 0.572 | 0.683 | 0.495 | - | *rs1059369AT* | -0.015 | 0.478 | -0.031 | 0.975 | - |
| *rs888663TT* | 0.420 | 0.548 | 0.766 | 0.444 | - | *rs1059369TT* | -0.080 | 0.462 | -0.170 | 0.865 | - |
| *Primiparity (Yes)* | -0.554 | 0.218 | -2.542 | **0.011*** | 0.575 (0.374-0.879) | *Primiparity (Yes)* | -0.523 | 0.218 | -2.399 | **0.016*** | 0.592 (0.385-0.907) |
|  |  |  |  |  |  |  |  |  |  |  |  |
| *(Intercept)* | -2.482 | 0.792 | -3.133 | **0.002**** | 0.084 (0.016-0.370) | *(Intercept)* | -1.496 | 0.649 | -2.305 | **0.021*** | 0.224 (0.062-0.794) |
| *rs888663GT* | 0.966 | 0.656 | 1.472 | 0.141 | - | *rs1059369AT* | 0.008 | 0.485 | 0.016 | 0.987 | - |
| *rs888663TT* | 0.923 | 0.635 | 1.454 | 0.146 | - | *rs1059369TT* | -0.073 | 0.469 | -0.156 | 0.876 | - |
| *Age (years)* | 0.061 | 0.018 | 3.446 | **0.001***** | 1.063 (1.027-1.100) | *Age (years)* | 0.059 | 0.018 | 3.353 | **0.001***** | 1.061 (1.025-1.099) |

Abbreviations: GAS, gestational age at sampling; CI, confidence intervals; OR, odds ratio; GH, gestational hypertension.

Significant *P* values are in bold.
