# Supplementary material for: Functional characterization of the GWAS lead SNP rs888663 and effects of GDF15 SNPs on GDF15 levels in gestational hypertension and preeclampsia
Source: Mol Biol Rep. 2026 Mar 7;53(1):476. doi: 10.1007/s11033-026-11629-w (PMC12967388; doi:10.1007/s11033-026-11629-w)
Supplement: Supplementary file 5 — Supplementary Material 5 [file 11033_2026_11629_MOESM5_ESM.docx]

**Supplementary Table 4.** Multivariate logistic regression analysis adjusted for independent variables in Preeclampsia (PE).

| *Logistic model PE* | *Estimate* | *Std. Error z* | *z value* | *Pr(>\|z\|)* | *OR (95% CI)* |  | *Estimate* | *Std. Error z* | *z value* | *Pr(>\|z\|)* | *OR (95% CI)* |
| --- | --- | --- | --- | --- | --- | --- | --- | --- | --- | --- | --- |
| *(Intercept)* | -5.710 | 1.013 | -5.636 | **<0.001** | 0.003 (0.001-0.023) | *(Intercept)* | -5.198 | 0.911 | -5.705 | **<0.001** | 0.006 (0.001-0.031 |
| *rs888663GT* | 0.070 | 0.635 | 0.110 | 0.913 | - | *rs1059369AT* | -0.514 | 0.521 | -0.987 | 0.324 | - |
| *rs888663TT* | 0.217 | 0.604 | 0.358 | 0.720 | - | *s1059369TT* | -0.420 | 0.499 | -0.842 | 0.340 | - |
| *Age (years)* | 0.050 | 0.019 | 2.598 | **0.009**** | 1.050 (1.013-1.091) | *Age (years)* | 0.049 | 0.019 | 2.590 | **0.010**** | 1.051 (1.012-1.091) |
| *BMI (kg/m²) during pregnancy* | 0.140 | 0.023 | 5.959 | **<0.001** | 1.145 (1.100-1.206) | *BMI (kg/m²) during pregnancy* | 0.142 | 0.024 | 6.020 | **<0.001** | 1.153 (1.103-1.210) |
|  |  |  |  |  |  |  |  |  |  |  |  |
| *(Intercept)* | -1.799 | 0.676 | -2.661 | **0.008**** | 0.166 (0.042-0.618) | *(Intercept)* | -1.422 | 0.621 | -2.291 | **0.022*** | 0.241 (0.070-0.811) |
| *rs888663GT* | 0.122 | 0.557 | 0.218 | 0.827 | - | *rs1059369AT* | -0.179 | 0.484 | -0.369 | 0.712 | - |
| *rs888663TT* | 0.190 | 0.528 | 0.359 | 0.720 | - | *s1059369TT* | -0.233 | 0.468 | -0.498 | 0.618 | - |
| *Age (years)* | 0.062 | 0.018 | 3.513 | **0.001***** | 1.064 (1.028-1.102) | *Age (years)* | 0.061 | 0.018 | 3.492 | **0.001***** | 1.063 (1.028-1.101) |
|  |  |  |  |  |  |  |  |  |  |  |  |
| *(Intercept)* | -4.715 | 0.9142 | -5.158 | **<0.001** | 0.009 (0.001-0.051) | *(Intercept)* | -4.161 | 0.795 | -5.233 | **<0.001** | 0.016 (0.003-0.071) |
| *rs888663GT* | 0.287 | 0.6184 | 0.465 | 0.642 | - | *rs1059369AT* | -0.483 | 0.515 | -0.939 | 0.348 | - |
| *rs888663TT* | 0.274 | 0.5910 | 0.464 | 0.643 | - | *s1059369TT* | -0.342 | 0.493 | -0.694 | 0.488 | - |
| *BMI (kg/m²) during pregnancy* | 0.147 | 0.0231 | 6.366 | **<0.001** | 1.158 (1.109-1.214) | *BMI (kg/m²) during pregnancy* | 0.150 | 0.023 | 6.416 | **<0.001** | 1.162 (1.112-1.219) |

Abbreviations: GAS, gestational age at sampling; CI, confidence intervals; OR, odds ratio; PE, preeclampsia. Significant *P* values are in bold.
