# Supplementary material for: Functional characterization of the GWAS lead SNP rs888663 and effects of GDF15 SNPs on GDF15 levels in gestational hypertension and preeclampsia
Source: Mol Biol Rep. 2026 Mar 7;53(1):476. doi: 10.1007/s11033-026-11629-w (PMC12967388; doi:10.1007/s11033-026-11629-w)
Supplement: Supplementary file 6 — Supplementary Material 6 [file 11033_2026_11629_MOESM6_ESM.docx]

**Supplementary Table 5.** Clinical and demographic characteristics for subjects in the healthy pregnant, preeclampsia and gestational hypertension groups whose plasma GDF15 levels were measured.

| ***Parameters*** | ***Healthy pregnant***  ***(n = 68)*** | ***Preeclampsia***  ***(n = 72)*** | ***p^a^*** | ***Gestational hypertension***  ***(n = 63)*** | ***p^b^*** | ***p^c^*** |
| --- | --- | --- | --- | --- | --- | --- |
| Age (years) | 24.4 ± 0.7 | 27.2 ± 0.8 | **0.015** | 26.7 ± 0.9 | 0.100 | 0.332 |
| Ethnicity (% White) | 73.33 | 65.28 | 0.684 | 70.97 | 1.000 | 0.787 |
| Current smokers (%) | 12.07 | 6.95 | 0.383 | 14.29 | 0.796 | 0.266 |
| BMI (kg/m²) before pregnancy | 24.60 ± 0.7 | 29.90 ± 1.05 | **0.000** | 30.87 ± 1.1 | **0.000** | 0.528 |
| BMI (kg/m²) during pregnancy | 29.3 ± 0.7 | 33.95 ± 0.8 | **0.000** | 35.58 ± 0.9 | **0.000** | 0.177 |
| SBP (mmHg) | 110.5 ± 1.4 | 138.3 ± 2.9 | **0.000** | 130.8 ± 2.2 | **0.000** | **0.004** |
| DBP (mmHg) | 71.1 ± 1.2 | 87.4 ± 1.6 | **0.000** | 83.1 ± 1.4 | **0.000** | **0.039** |
| HR (beats per min) | 81.1 ± 1.0 | 80.7 ± 0.8 | 0.864 | 81.4 ± 1.1 | 0.991 | 0.852 |
| Fasting glucose (mg/dL) | 74.3 ± 2.0 | 84.3 ± 2.1" | **0.002** | 87.1 ± 3.3 | **0.003** | 0.419 |
| Hb (mg/dL) | 11.8 ± 0.2 | 11.6 ± 0.2" | 0.805 | 12.0 ± 0.2" | 0.218 | 0.258 |
| Hct (%) | 35.6 ± 0.7 | 34.8 ± 0.6 | 0.690 | 36.1 ± 0.5 | 0.426 | 0.147 |
| Creatinine (mg/dL) | 0.7 ± 0.1 | 0.7 ± 0.0 | 0.367 | 0.6 ± 0.0 | 0.056 | **0.017** |
| 24h Pr (mg per 24h) | ND | 1061 ± 175.6 | **-** | 154.9 ± 12.5 | **-** | **0.000** |
| Primiparity (%) | 52.5 | 29.6 | 0.101 | 36.5 | 0.327 | 0.604 |
| GAD (weeks) | 39.96 ± 0.2 | 36.64 ± 0.5 | **0.000** | 38.83 ± 0.3 | **0.001** | **0.001** |
| Newborn weight (g) | 3395 ± 59.3 | 2758 ± 111.4 | **0.000** | 3195 ± 84.4 | 0.153 | **0.006** |
| GAS (weeks) | 36.76 ± 0.4 | 34.10 ± 0.6 | **0.003** | 35.11 ± 0.7 | 0.376 | 0.078 |
| Early-onset PE (%) | ND | 19.5 |  | ND |  |  |

Abbreviations: BMI, body mass index; DBP, diastolic blood pressure; GAD, gestational age at delivery; GAS, gestational age at sampling; Hb, hemoglobin concentration; Hct, hematocrit; HR, heart rate; ND, not determined (however, negative dipstick test); SBP, systolic blood pressure; 24-h Pr, 24-h proteinuria. Values are the mean ± s.e.m.

*p^a^*^,^ healthy pregnant vs. preeclampsia.

*p^b^,* healthy pregnant vs. gestational hypertension.

*p^c^,* preeclampsia vs. gestational hypertension

Significant *p* values are in bold.
